# Imported COVID-19: The Challenges of Emigration Screening

**DOI:** 10.1017/dmp.2021.21

**Published:** 2021-03-30

**Authors:** Rujittika Mungmunpuntipantip, Viroj Wiwanitkit

**Affiliations:** 1 Private Academic Consultant, Bangkok Thailand; 2 Dr DY Patil University, Pune, India; 3 Hainan Medical University, Haikou, China

**Keywords:** mass screening, infection control, infectious disease medicine

COVID-19 is an important new coronavirus infection. The disease it causes has been identified in more than 60 countries.^1^ Indochina was the second area with an identified case in the timeline of the COVID-19 pandemic and Thailand is the first area reporting the case outside China.^2^ Screening for disease at international border posts was a national public health policy against COVID-19 implemented from January 2020 onward. The screening had apparent limitations,^2^ as other countries still reported detection of COVID-19 in travelers from Indochina.^3^ In this letter, the authors discuss the topic of COVID-19 exported to other countries, which can illustrate the challenges of emigration screening. According to data from local Immigration Bureau, 3338710 foreigners were screened for COVID-19 at the international emigration post. Regarding exportation of disease, at least 3 patients who had a history of traveling have been confirmed to have COVID-19. The rate of undetected COVID-19 cases at the emigration international border post is therefore 9 x 10^−5^% (95% confidence interval between 3 x 10^-5^% and 2.8 x 10^−4^%). In the worst case scenario, 3 out of 1000000 travelers might carry COVID-19 to their next destination. Entrance or immigration screening is usually intensive, while exit or emigration screening is less strict. A good disease screening system for both immigration and emigration can promote early detection and prevent the international spread of COVID-19.
